# Plasmonic Circular Dichroism in Chiral Gold Nanowire Dimers

**DOI:** 10.3390/molecules27010093

**Published:** 2021-12-24

**Authors:** Daniele Toffoli, Marco Medves, Giovanna Fronzoni, Emanuele Coccia, Mauro Stener, Luca Sementa, Alessandro Fortunelli

**Affiliations:** 1 Dipartimento di Scienze Chimiche e Farmaceutiche, Università di Trieste, Via Giorgieri 1, 34127 Trieste, Italy; toffoli@units.it (D.T.); marcomedves@virgilio.it (M.M.); fronzoni@units.it (G.F.); ecoccia@units.it (E.C.); 2 CNR-ICCOM & IPCF, Consiglio Nazionale delle Ricerche, Via Giuseppe Moruzzi 1, 56124 Pisa, Italy; luca.daumann@gmail.com

**Keywords:** plasmon, circular dichroism, nanoplasmonics, metal clusters

## Abstract

We report a computational study at the time-dependent density functional theory (TDDFT) level of the chiro-optical spectra of chiral gold nanowires coupled in dimers. Our goal is to explore whether it is possible to overcome destructive interference in single nanowires that damp chiral response in these systems and to achieve intense plasmonic circular dichroism (CD) through a coupling between the nanostructures. We predict a huge enhancement of circular dichroism at the plasmon resonance when two chiral nanowires are intimately coupled in an achiral relative arrangement. Such an effect is even more pronounced when two chiral nanowires are coupled in a chiral relative arrangement. Individual component maps of rotator strength, partial contributions according to the magnetic dipole component, and induced densities allow us to fully rationalize these findings, thus opening the way to the field of plasmonic CD and its rational design.

## 1. Introduction

Among the wide variety of phenomena which emerge at the nanoscale régime, the localized surface plasmon resonance (LSPR), typical of nanostructured metals, plays a major role in nanotechnology due to its ability of focusing the electromagnetic field with a high energy density in a small region of space [1,2,3]. This effect has direct application in enhanced spectroscopic techniques, such as surface enhanced Raman spectroscopy [4], and in single-molecule spectroscopy [5,6]. Additionally, the possibility of having LSPR exhibiting chiral features is extremely appealing, because it would allow one to enhance the specificity and selectivity of sensing devices, especially in the biological applications [7]. It is worth noting that, while conventional plasmons in extended systems are well understood as collective motions of conduction band electrons, for finite systems such as nanoclusters and nanowires, their nature is still debated [8,9,10,11,12]. The picture is even less clear in the field of chirality, as experiments are here much more difficult to conduct and interpret, and less conclusive. For all these reasons, contribution from theoretical insight and understanding is dramatically required.

Chiral systems generally give a non-zero circular dichroism (CD) signal. The CD response is routinely employed to study biomolecules, while frontier applications are in the emerging field of chiral sensing [13]. Managing the CD signal is challenging due to its intrinsic weakness, usually five orders of magnitude less intense than the corresponding absorption signal, as a consequence of the electric-dipole/magnetic-dipole scalar product that governs the CD intensity according to the Rosenfeld equation [14]. For this reason, the amplification of the CD signal in plasmonic systems is quite intriguing, and many attempts have been reported along this research direction [15,16,17,18]. It is convenient to classify plasmonic CD into structural and induced CD [18], according to its physical origin. A chiral plasmonic metal cluster or nanostructure gives rise to structural plasmonic CD [19], while a chiral arrangement of non-chiral systems promotes an induced plasmonic CD. Although induced plasmonic CD is easier to obtain at the experimental level, [20] the field of structural chiral plasmonics, although still in its infancy, offers very promising possibilities, for example, chiral growth promoted by hot electron mechanisms in nanocrystals [21] or hot-electron transfer [22]. Despite the interest in these new developments, at present, at the experimental level, only modest plasmonic CD enhancements have been observed [18]. We believe that a rationalization of both structural and induced plasmonic CD in terms of quantum mechanics is needed to allow understanding the issues limiting experimental observations, the design of optimal systems, and the ensuing exploitation of this phenomenon. To this aim, some of us recently studied the CD of a series of chiral plasmonic gold nanowires by means of time dependent density functional theory (TDDFT) [23]. As a main outcome of this work, we found that chiral linear gold nanowires do not give rise to a plasmonic CD, notwithstanding the presence of a very strong and sharp plasmonic resonance in absorption. In contrast, a very strong plasmonic CD was predicted when the chiral nanowires were no longer linear, but assumed a helical shape winding around the external surface of a cylinder. An analysis based on the individual component mapping of the rotator strength (ICM-RS) [24] allowed us to ascertain that, for linear nanowires, the absence of CD in correspondence of the plasmon absorption is due to a destructive interference among huge contributions of opposite sign. This finding then triggered the present analysis, where we now try to overcome and bypass the destructive interference phenomenon and also recover a plasmonic CD in linear nanowires (that are much easier to synthetize) by coupling two chiral nanowires. As we will detail below, the ICM-RS analysis in fact suggested that the nanowire/nanowire interaction can perturb the interference between positive and negative contributions to CD, thus greatly decreasing its destructive character, and therefore leading to plasmonic circular dicroism.

In the present work, we considered various coupling modes between the chiral nanowires: we started with two chiral nanowires coupled via an achiral relative arrangement (pure structural CD); then we considered two chiral nanowires coupled in a chiral relative arrangement (structural and induced CD). In the latter case, in order to identify the effect of pure induced CD, we also considered two achiral nanowires coupled in a chiral relative arrangement. We then showed that the nanowire coupling indeed produced the expected effect of strongly diminishing the destructive interference phenomenon, such that we predicted to the best of our knowledge for the first time an intense plasmonic CD also in linear nanowire systems. ICM-RS, partial contributions according to the magnetic dipole components, and induced densities were then exploited to fully rationalize our finding. In general, gold nanowires are systems which, besides chirality, are interesting for their properties, e.g., their propensity to form hybrid structures such as encapsulation in single wall carbon nanowires (SWCN) [25].

## 2. Discussion

In our previous work [23], the structure of the (5,3)NT nanowire was inspired by experimental work on the synthesis of helical gold multi-shell nanowires [26]. The starting point is given by the helical linear structure (constructed according to the prescription of Senger et al. [27]) that is intrinsically chiral. The calculated plasmon was very intense in absorption, but no CD signal was found at the energy of the plasmon resonance [23].

Since we found that the plasmon CD was suppressed by destructive interference, we suggested that a perturbation of the system might remove, at least partially, such destructive interference. In this work, we explored this idea considering the interaction between pairs of nanowires.

**Figure 1:**
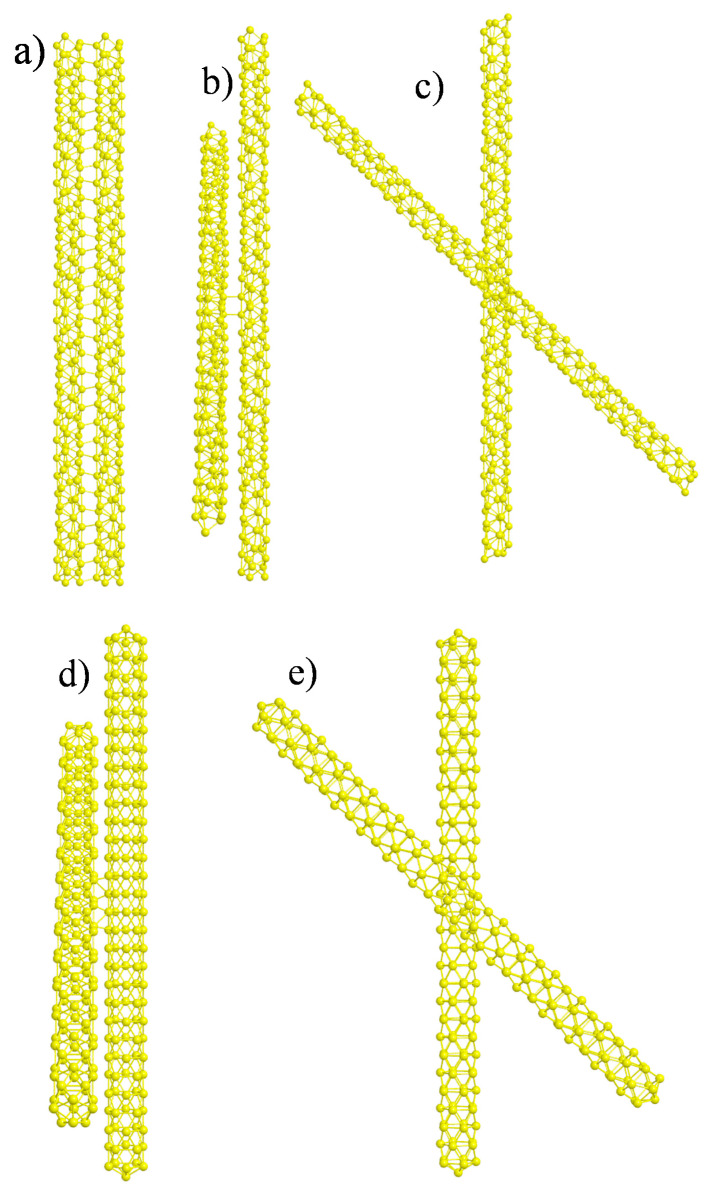
(**a**) Structure of a pair of interacting gold chiral nanowires with parallel axis so their arrangement is not chiral. (**b**) side view and (**c**) top view of a pair of interacting gold chiral nanowires with axis rotated by 45 degrees so their relative orientation is chiral. (**d**) side view and (**e**) top view of a pair of interacting gold achiral nanowires with axis rotated by 45 degrees so their relative orientation is chiral.

The simplest interaction between two nanowires is obtained by keeping their axis parallel to each other and changing only their relative distance. In Figure 1a, the geometry actually employed in the calculations is reported, where the original structure of the (5,3)NT taken from ref. [23] has been repeated two times in order to keep the C_2_ symmetry *z*-axis: we will refer to this system in the following as the parallel geometry. 

The distance between the two nanowires has been set to 2.88 Å, equal to the bulk gold–gold interatomic distance. Note that, in this case, the two nanowires were both chiral but their relative orientation was achiral. The chosen distance between Au atoms corresponds to the distance of physical systems not otherwise constrained (the nanowires will tend to touch each other to minimize energy). We have also tried a larger distance between nanowires equal to 5 Å; however, in that case the nanowires were non-interacting and we did not obtain an appreciable difference in absorption and CD spectra with respect to a single nanowire. Distances between 2.88 Å and 5 Å were difficult to explore because the chemical bonds connecting the two nanowires were partially broken, making the SCF difficult to converge.

In Figure 1b,c another geometric configuration is reported, which is derived from the previous parallel geometry obtained via a rotation of only one of the two nanowires by 45 degrees around the C_2_ z-axis: we will refer to this system as the rotated geometry. In Figure 1 we have reported the same system viewed from two different perspectives: in the side view (b), the chemical bonds connecting the two nanowires are visible, while in top view (c) it is possible to appreciate the relative rotation of the two nanowires. In this case, the relative orientation itself is chiral; in fact, in the present relative orientation, we do not have any symmetry plane and a chiral system is obtained. We may therefore distinguish between structural chirality (of the single nanowire) and induced chirality (by the relative orientation between the two objects). In order to distinguish even more clearly the two effects we have also considered a chiral relative orientation of two achiral nanowires, like in Figure 1d,e. In this case we first built an achiral nanowire with the same size of the chiral one (152 gold atoms), whose structure has been generated starting from the Au_12_ icosahedral cluster with a gold–gold interatomic distance of 2.88 Å, adding 14 equatorial ribbons of Au_10_ units, obtaining finally the Au_152_ cluster with D_5d_ symmetry. Then two of such clusters were paired in the same way as the rotated geometry of the previous Figure 1b,c. This new configuration was reported in Figure 1d,e and will be referred to as *rotated achiral*; note that, in this case, only induced plasmonic CD is expected. 

**Figure 2:**
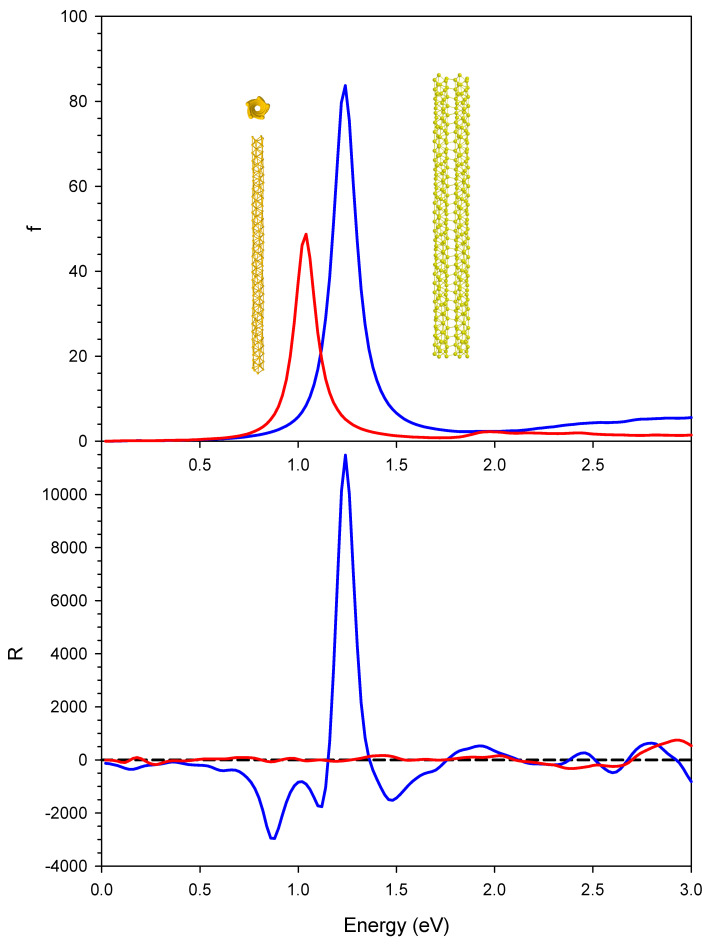
Photoabsorption (upper panel) and CD (lower panel) for the single chiral nanowire (red lines) and the pair of interacting gold chiral nanowires with parallel axis (blue line). Oscillator strengths are given in atomic units, while R is given in 10^−40^ esu^2^·cm^2^.

**Figure 3:**
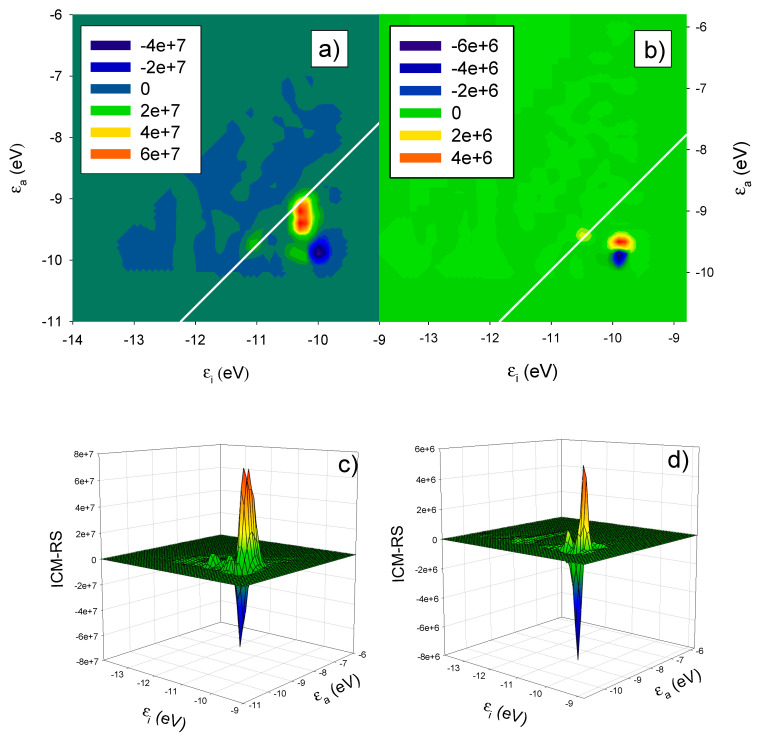
ICM-RS plots relative to the y component taken at the energy corresponding to the maximum CD: 1.04 eV for the single nanowire in panels (**a**,**c**); 1.24 eV for the pair of interacting gold chiral nanowires with parallel axis in panels (**b**,**d**) as in Figure 1. ε_i_ and ε_a_ are energies of occupied and virtual orbitals, respectively.

The photoabsorption (upper panel) and CD (lower panel) for the single chiral nanowire (red lines) and the pair of interacting gold chiral nanowires with parallel axis (blue line) are reported in Figure 2. The single chiral nanowire results have been taken from our previous work [23]. The effect of coupling on the photoabsorption is an intensity enhancement with the absorption approximately doubled in the two-nanowire systems, and a blue shift from 1.04 eV to 1.24 eV of the plasmon peak. These results can be rationalized in terms of plasmon coupling: the induced dipoles on the two nanowires are parallel; as a consequence, the coupled plasmon energy is increased due to their repulsive interaction. In contrast, the effect of nanowire coupling on the CD is striking: while the single nanowire gives negligible CD at the plasmon energy, the pair with parallel axis gives a huge positive contribution, with a peak value exceeding 10,000 × 10^−40^ esu^2^·cm^2^, i.e., an increase of four orders of magnitude of the CD signal with respect to the single-nanowire system. This value is of the same order of magnitude as that obtained for the helical nanowires [23], which reached a maximum around 40,000 × 10^−40^ esu^2^·cm^2^. In the Figure 3 we have reported the ICM-RS plots of both present pair of interacting nanowires (boxes (a) and (c)) as well as those of the single chiral nanowire (boxes (b) and (d)) taken from our previous work [23]. Moreover, we have generated both 2D (boxes (a) and (b)) as well as 3D plots (boxes (c) and (d)) in order to have a more direct visualization of the effects. All the details regarding the definition and calculation of the ICM-RS plots have been reported in Appendix A.2 of the Appendix A of the present work. Such plots consist of decomposing the rotator strength (R) of a given transition in its components in terms of occupied-virtual pairs; on the x and y axis, the occupied and virtual orbital energies are considered. The presence of a ‘spot’ indicates that the orbital pair that had the corresponding energy is involved in the transition. In 2D, the ‘intensity’ of the involvement is given by a colour scale; for 3D plots, the ‘intensity’ corresponds to the scale of the z axis. As observed previously and considering the present Figure 3b,d, the negligible CD of the single chiral nanowire is a consequence of a destructive interference of two opposite and large contributions. These opposite (positive and negative) contributions in the ICM-RS spectrum of the (5,3) nanowire are individually very large but are practically equal in absolute value. They thus cancel each other almost perfectly, producing a nearly zero CD spectrum. This suggested that by perturbing the system with a proper coupling, it should be possible to remove, at least partially, such a destructive interference, allowing the manifestation of a plasmonic CD. The present results fully confirm this hypothesis: here we have demonstrated that the coupling between a pair of nanowires is sufficient to allow a partial suppression of the destructive interference phenomenon. Indeed, in Figure 3a,d, we report the ICM-RS plots of the parallel pair taken at the energy corresponding to the maximum dichroism. Only the y dipole component was considered (along the direction of maximum nanowire length), the other components being negligible. It is evident that there was still destructive interference, since there were regions with the opposite sign, but now the positive region was wider and more intense than the negative one, such that there was only a partial cancellation. However, this also shows that the destructive interference had been only partially removed, and that there was still wide room for further increasing the dichroism, suggesting a promising path for future work. 

**Figure 4:**
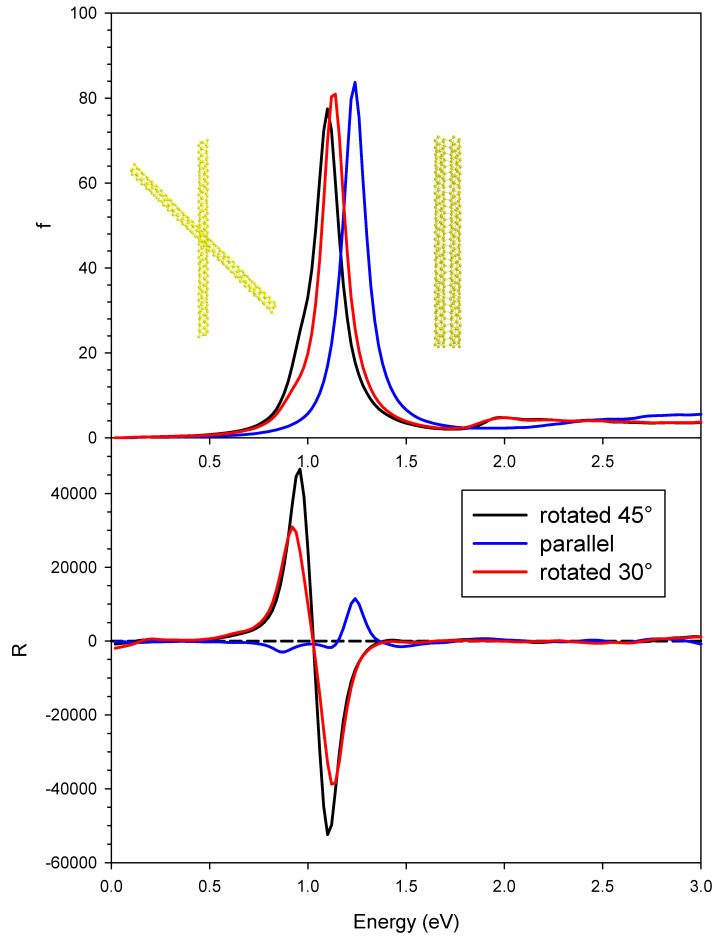
Photoabsorption (upper panel) and CD (lower panel) for the pair of interacting gold chiral nanowires with parallel axis (blue line) and rotated axis by 45° (black line) and 30° (red line). Oscillator strengths are given in atomic units, while R is given in 10^−40^ esu^2^·cm^2^.

We then analyzed the rotated geometry described in Figure 1b,c, whose photoabsorption and dichroism are reported in Figure 4, together with the results of the parallel geometry for comparison. The plasmon in absorption of the rotated systems displayed a red shift with respect to the parallel one, going from 1.24 eV to 1.10 eV, while the oscillator strengths showed only a modest decrease. The red shift could be easily rationalized if we described the plasmon as the sum of the dipolar plasmons of the individual nanowires. The destabilizing interaction between the dipoles was reduced in the rotated systems, producing a red shift. We tried to study the effect of varying the angle between the nanowires on the spectral features. This was not a straightforward task, since the mutual orientation between the two nanowires had to allow a suitable formation of chemical bonds between them; if such bonds were too deformed, the SCF procedure would not converge, hampering such analysis. The only angle we were able to consider was 30°; we have reported in Figure 4 the corresponding plots as a red line. Such results (both in terms of photoabsorption and CD) were very similar to those obtained by a rotation of 45°, lying in between the 45° and the parallel ones. For this reason, we limit further discussion to the geometry rotated by 45°.

**Figure 5:**
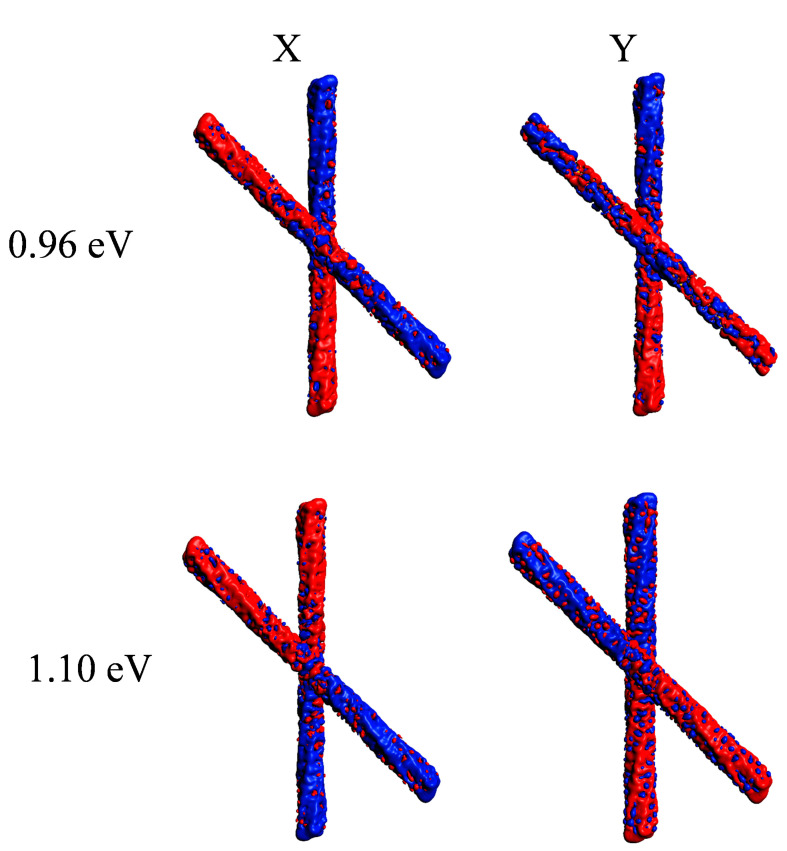
Induced density plots relative to the x and y components taken at the energy corresponding to the maximum CD (0.96 eV) and the minimum CD (1.10 eV) of a pair of interacting gold chiral nanowires with axis rotated by 45 degrees as in Figure 1.

In contrast, the difference between the CD profiles is dramatic: while the parallel system gives in practice only one very strong positive feature up to 10,000 × 10^−40^ esu^2^·cm^2^, the one rotated by 45° gave a pair of strong peaks with opposite sign, separated by only 0.14 eV, with rotator strength up to ±50,000 × 10^−40^ esu^2^·cm^2^. This finding suggests that, in this case, induced plasmonic CD was stronger than structural plasmonic CD, analogously to what was reported in our previous work for the comparison between linear and helical nanowires [23]. It is hard to say if this is a general behavior or one specific to the present systems. In fact, it is worth noting that the structural plasmonic observed in the parallel situation kept a large amount of destructive interference; therefore, it is still possible that more effective coupling between nanowires may remove further the destructive character giving rise to much higher structural plasmonic CD. Induced densities of the rotated geometry at the two energies corresponding to the maximum and minimum CD, respectively 0.96 and 1.10 eV, are reported in Figure 5. In both cases, the induced density displays a clear dipolar shape for each individual nanowire, typical of a plasmon. However, at lower energy, the individual dipoles of the wires displayed opposite direction, corresponding to a negative scalar product; at higher energy, the dipoles had the same direction.

**Figure 6:**
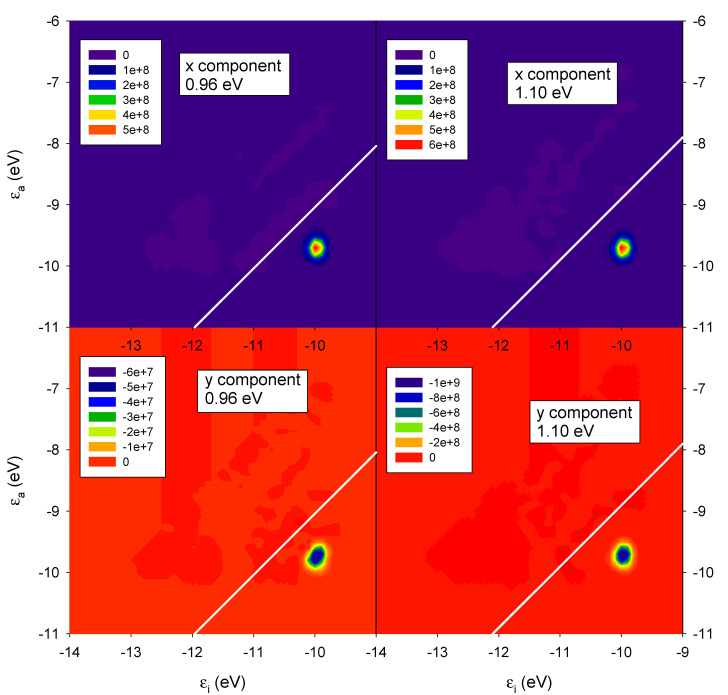
ICM-RS plot relative to the x and y components taken at the energy corresponding to the maximum CD (0.96 eV) and the minimum CD (1.10 eV) of a pair of interacting gold chiral nanowires with axis rotated by 45 degrees as in Figure 2. ε_i_ and ε_a_ are energies of occupied and virtual orbitals, respectively.

**Figure 7:**
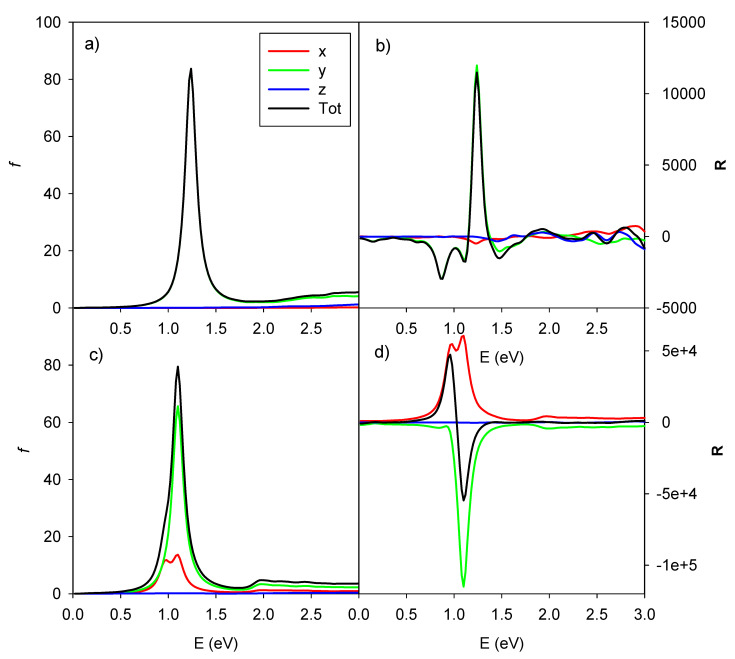
Dipole components partial contributions of photoabsorption (left panels (**a**,**c**)) and CD (right panels (**b**,**d**)) for the pair of interacting gold chiral nanowires with parallel axis (upper panels (**a**,**b**)) and rotated axis (lower panels (**c**,**d**)). Oscillator strengths are given in atomic units, while R is given in 10^−40^ esu^2^·cm^2^, x, y, z and total contributions are in red, green, blue and black lines, respectively.

In order to have a better understanding of the phenomenon, we show in Figure 6 the ICM-RS of the two CD peaks at 0.96 eV (left boxes) and 1.10 eV (right boxes). Both x dipole component (upper boxes), as well y dipole component (lower boxes), are reported. Such ICM-RS plots are quite different from that of the parallel system (Figure 3). Indeed, we did not observe any destructive interference for the rotated geometry for x and y components of the dipole. In fact, for both energies, the x component was positive and the y component was negative for all of the spots on the 2D ICM-RS plot. More precisely, at 0.96 eV, the x component was more positive than the negative y component, a situation that was reversed at 1.10 eV; here, the order of magnitude of the x component did not change, while the negative y components increased by two orders of magnitude and became preponderant. Since the present ICM-RS analysis suggests that the mutual interplay between individual dipole components was fundamental to rationalize the specific CD behavior, in Figure 7 we have considered, for both parallel and rotated geometries, the photoabsorption and CD partial profiles from the dipole components. For the parallel geometry the situation was obviously quite simple: only the y component played a fundamental role for both photoabsorption and CD, the y axis being along the nanowires axis. For the rotated geometry, instead, the situation was more interesting: the total photoabsorption peak was contributed essentially by both x and y components; however, while the y component had a single maximum at 1.10 eV, the x component displayed two maxima at 0.98 and 1.10 eV. Consistently with what was already found in the ICM-RS analysis the x component had very similar values at the two energies, while the y component increased by a factor of four, going from 0.98 eV to 1.10 eV. In practice, the feature at 0.98 eV almost disappeared in the total profile; thus, only a sketched shoulder can be hardly seen in the left side of the peak. Passing to the CD, the partial profiles display similar shape (two maxima for the x dipole and one maximum for the y dipole); however, the y component was negative, which explains the observed behavior in the total profile. 

**Figure 8:**
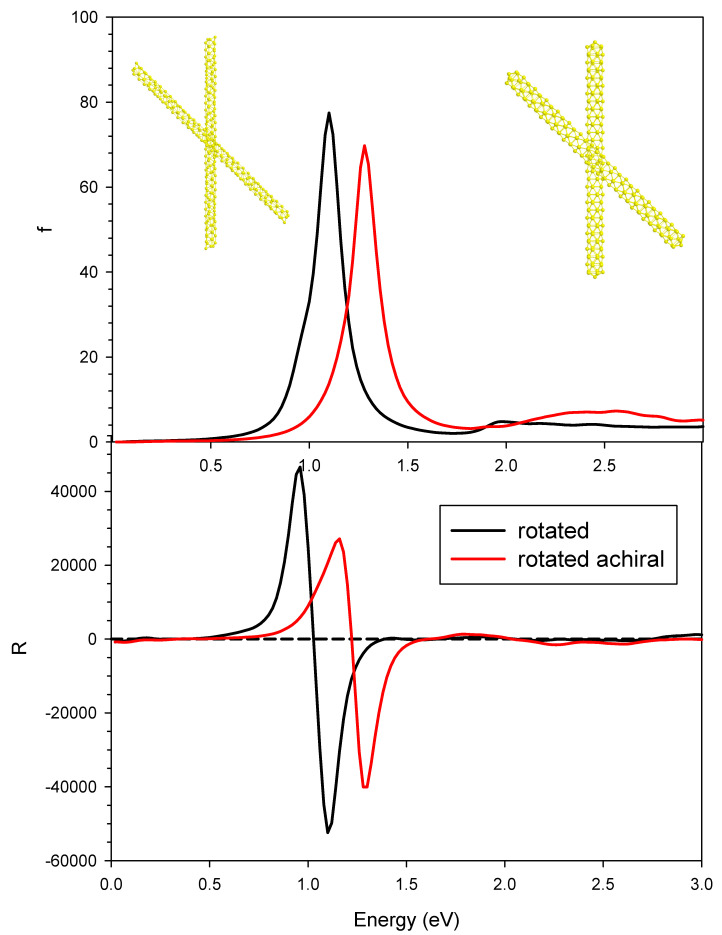
Photoabsorption (upper panel) and CD (lower panel) for the pair of interacting gold chiral nanowires with rotated axis (black line) and achiral nanowires with rotated axis (red line). Oscillator strengths are given in atomic units, while R is given in 10^−40^ esu^2^·cm^2^.

In order to better identify the role of the induced plasmonic CD, we report in Figure 8 the photoabsorption and the CD of the rotated achiral system, whose geometry is reported in Figure 1d,e, together with the profiles of the rotated chiral system for comparison. We observed a blue shift for both photoabsorption and CD profiles; the photoabsorption was slightly attenuated, while, for CD, the weakening seemed more pronounced. The nanowires that constitute this system are achiral, so the CD is purely an induced one: its maximum and minimum values of 30,000 × 10^−40^ and −40,000 × 10^−40^ esu^2^·cm^2^ are roughly a factor of three larger than the pure structural CD reported in the previous Figure 2 for the parallel geometry. We may conclude this analysis by saying that, at least for the system considered in the present study, the strength of the induced CD was roughly three times the structural one, and these effects sum up when both of them are present in the same system. 

## 3. Conclusions

In this work, TDDFT simulations were performed on a series of dimers (pairs) of chiral gold nanowires to explore whether an enhancement of circular dichroism at the plasmon resonance is possible through a coupling between nanostructures.

We find that, when two chiral nanowires are coupled in an achiral relative arrangement, a strong enhancement of the plasmonic CD is predicted by theory with respect to the separate nanowires, which individually did not give any appreciable plasmonic CD. This demonstrates that coupling between two ‘inactive because of destructive interference’ chiral plasmonic systems could still give strong structural CD. We fully rationalized this finding via an analysis of ICM-RS plots. The enhancement is even more pronounced when two chiral nanowires are coupled in a chiral relative arrangement, because, in this configuration, the structural and induced effects sum up to give the largest effect. In order to distinguish between structural CD and induced CD we also considered two achiral nanowires coupled in a chiral relative arrangement, in which case the plasmonic CD was purely induced. From such analysis, at least for the gold clusters and the configurations considered in this work, we found that the induced plasmonic CD is somewhat more intense than the structural one and the two effects sum up when they are simultaneously present. Further studies to demonstrate the generality of the present conclusions would be desirable, especially to identify which are the most effective coupling between chiral systems to produce the highest plasmonic CD phenomenon. In this respect we can, e.g., hypothesize that the addition of ligands adsorbed on the wires, thus locally perturbing their wave functions in a proper way, could be a possible (and technically easier to materialize) alternative to obtain a strong plasmonic CD with respect to coupling pairs of nanowires as in the present work.

## Data Availability

The data presented in this study are available on request from the corresponding author.

## Sample Availability

Samples of the compounds are not available from the authors.

## Appendix A. Theoretical Method

The computational study of CD in plasmons is challenging due to the large size of the metal clusters involved and the chirality, which implies very low symmetry (if any), of the system. Therefore, very accurate (explicitly correlated) ab-initio methods are ruled out, while the density functional theory (DFT) and TDDFT represent reliable options. The first CD calculation at the TDDFT level has been done by Autschbach and Ziegler [28] using the Casida scheme [29], which consists of diagonalizing a large matrix, with dimensions corresponding to the number of occupied-virtual orbitals pairs. This method is very efficient to investigate the lowest part of the spectrum, a situation which is not effective for nanoclusters in which a large number of roots are needed to cover the optical region. For this reason, a TDDFT algorithm which avoids large matrix diagonalization has been implemented [30], named polTDDFT, and extended to the calculation of the CD spectrum [31].

### Appendix A.1. The Complex Polarizability Method

The reader is referred to the original work for a detailed description of the algorithm [30], together with its implementation in the ADF program [32].

In practice, the photoabsorption spectrum *σ**(**ω**)* is calculated point by point, from the imaginary part of the dynamical polarizability *α**(**ω**)*:

(A1) σ(ω)=4πωcIm[α(ω)]

This expression is of practical interest when the polarizability is calculated for complex frequency, i.e., *ω* *=* *ω**_r_* *+ i**ω**_i_*, where the real part *ω**_r_* is the scanned photon frequency (energy) and *ω*_i_ is the imaginary part that corresponds to a broadening of the discrete lines and can be interpreted as a pragmatic inclusion of the excited states’ finite lifetime. The complex dynamical polarizability is calculated by solving the following non-homogeneous linear system:

(A2) [S−M(ω)]b=d

In Equation (A2) **S** is the overlap matrix between fitting functions, **b** is the unknown vector with the expansion coefficients b_µ_(*ω*) of the induced density ρ^(1)^_z_, **d** is the frequency dependent vector corresponding to the known non-homogeneous term, and finally the elements of the frequency dependent matrix **M** are:

(A3) $$M_{\mu \nu }=\langle f_{\mu }|\chi_{KS}\left(\omega \right)K|f_{\nu }\rangle$$

In Equation (A3), χ_KS_ refers to the Kohn-Sham frequency-dependent dielectric function and *K* to the kernel.

The original characteristic of the polTDDFT method is the introduction of a simple approximation which enables the construction of **M**(ω) as a linear combination of frequency independent matrices **G**^k^ with frequency dependent coefficients s_k_(ω), with the following expression:

(A4) $$M\left(\omega \right)=\sum_{k}s_{k}\left(\omega \right)G^{k}$$

With this idea, a set of matrices {**G**^k^} was calculated and stored only once at the beginning. Then the matrix **M**(*ω*) was calculated very rapidly at each photon energy ω, as a linear combination of the {**G**^k^} matrices with the following coefficients:

(A5) $$s_{k}\left(\omega \right)=\frac{4{\bar{E}}_{k}}{\omega^{2}-{\bar{E}}_{k}^{2}}$$

where in Equation (A5) ${\bar{E}}_{k}$ refers to the centre of the interval which discretizes the excitation energy variable and in the original formulation corresponds to the difference between virtual and occupied orbital energies: $\varepsilon_{a}-\varepsilon_{i}$.

In order to introduce the complex polarizability method to calculate the CD of large clusters, we briefly recall the basic theory of CD. For a molecule with fixed orientation, the CD of an electronic transition from the ground state |0 to the *n*-th excited state |n corresponds to the difference between the absorbance of left and right circularly polarized light, which propagates along the X direction as follows [33]:

(A6) CD=AL−AR=2γIm(〈0|μY|n〉〈n|mY|0〉+〈0|μZ|n〉〈n|mZ|0〉)

where in (6) **μ** and **m** are the electric dipole and magnetic dipole moment operators and *γ* is a constant.

In solution or in the gas phase molecules are randomly oriented, thus Equation (A6) must be rotationally averaged, and the Rosenfeld equation is obtained:

(A7) CD=43γIm(〈0|μ|n〉⋅〈n|m|0〉)

The rotatory strength, *R*_0*n*_, is therefore defined as follows:

(A8) R0n=Im(〈0|μ|n〉⋅〈n|m|0〉)

To calculate *R_0n_* by the complex polarizability algorithm [30], it is convenient to consider the dipole moment induced by an electromagnetic field [34]:

(A9) $$\mu_{u}^{\prime }=\sum_{v}\alpha_{uv}E_{v}-\sum_{v}\frac{\beta_{uv}}{c}\frac{\partial B_{v}}{\partial t}.$$

In Equation (A9) *E_v_* and *B_v_* are the electric and magnetic field components, *c* is the speed of light, *α* is the dynamical polarizability tensor and *β* is the optical rotation tensor, which is related to the rotatory strength by the following sum over states (SOS) expression:

(A10) $$\bar{\beta }=\frac{1}{3}\sum_{u}\beta_{uu}=\frac{2c}{3}\sum_{n}\frac{R_{0n}}{\omega_{0n}^{2}-\omega^{2}}$$

In Equation (A10) *ω* is the photon energy and *ω*_0*n*_ corresponds to the |0→|n excitation energy. Therefore, it is convenient to extract *R*_0*n*_ from the *β* imaginary part as in conventional photoabsorption. From Equation (A9), *β* consists in the electric dipole moment induced by a time-dependent (TD) magnetic field and can be calculated by the following expression:

(A11) $$\beta_{zz}\left(\omega \right)=\left(-\frac{ic}{\omega }\right)\sum_{i}^{occ}\sum_{a}^{virt}\langle \phi_{i}|m_{z}|\phi_{a}\rangle {\bar{\bar{P}}}_{i}^{a}$$

with

(A12) $${\bar{\bar{P}}}_{i}^{a}=t_{k}\left(\omega \right)\left[\langle \phi_{a}|\mu_{z}|\phi_{i}\rangle +\sum_{\mu \tau }^{fit}{\left(A^{k}\right)}_{ia,\mu }^{+}L_{\mu \tau }b_{\tau }\right]$$

In Equations (A11) and (A12), the $A_{\mu ,ia}^{k}$ are integrals between the auxiliary fitting function *f*_µ_ and the product between the *i*-th occupied and the *a*-th virtual orbitals, $\varphi_{i}|\mu_{z}|\varphi_{a}$ and $\varphi_{i}|m_{z}|\varphi_{a}$ are the electric and magnetic dipole moment matrix elements respectively, between the same occupied-virtual (*ia*) orbitals pair, the matrix **L** is defined by Equation (28) of Ref. [31], *t_k_* is given by:

(A13) $$t_{k}\left(\omega \right)=\frac{1}{\omega -\omega_{0n}+i\varepsilon }+\frac{1}{\omega +\omega_{0n}+i\varepsilon }$$

and the vector **b** is the solution of the linear system (2).

In practice, the resolution of the TDDFT equations is recast to the linear system (2) (see Ref. [30] for a detailed description), which was already solved to calculate the photoabsorption, so the CD calculation is computationally irrelevant. It is worth noting that the linear system (2) was solved by employing the auxiliary density fitting functions as a basis set to represent vectors and matrixes. This means that the dimension of (2) was much smaller with respect to the Casida approach.

Equation (A2) was then solved point by point for each photon energy. Moreover, the real part of the photon energy was supplemented with a small imaginary part, thus generating a Lorentzian broadening of the discrete transition.

### Appendix A.2. Individual Component Maps of Rotatory Strength (ICM-RS) Analysis

Recently, an analysis tool of the absorption spectra derived from TDDFT simulation has been proposed [35], i.e., individual component maps of oscillatory strength (ICM-OS) plots, which allows one to investigate the connection between absorption and single-particle excitations (ICM-OS). This field is subject to intense research efforts, and there are currently many efforts to find a proper method to rationalize CD (or in general spectral features) obtained from response calculations [36].

The same approach has been extended from the oscillator strength to the rotator strength, so in an analogous way we defined individual component maps of rotatory strength (ICM-RS) plots [24] as analysis tools of chiro-optical linear response spectra derived from TDDFT simulations. Starting with the expression of rotator strength at each given frequency (z component) calculated as the imaginary part of the zz diagonal element of the circular dichroic tensor:

(A14) CDz(ω)=−3ε2Re(∑iocc∑avirt〈ϕi|mz|ϕa〉P¯¯ia[z])

where in Equation (A14) ε corresponds to the Lorentzian energy broadening, ${\bar{\bar{P}}}_{i}^{a}$ is the density-matrix element given by previous Equation (A12), due to the perturbation induced by the z-component of the electric dipole, and $\varphi_{i}|m_{z}|\varphi_{a}$ is the matrix elements of the magnetic dipole over a pair of occupied/virtual single-particle molecular orbitals. Then the plot of the individual $\varphi_{i}|m_{z}|\varphi_{a}{\bar{\bar{P}}}_{i}^{a}\left[z\right]$ components as functions of the single-particle energies of occupied (ε_i_) and virtual (ε_a_) orbitals is generated. ICM-RS (*ω*) plots allow one to visualize the source of chiral response in momentum space, including signed contributions, therefore highlighting cancellation terms that are ubiquitous and critical in chiral phenomena.

### Appendix A.3. Computational Details

The geometry of the single gold nanowire (5,3)NT has been taken from our previous work, see Figure 1 in Ref. [23]. The DFT calculations have been performed with a Triple Zeta plus Polarization (TZP) basis set of Slater Type Orbitals (STO) functions. The LB94 [37] exchange-correlation (XC) potential with the correct asymptotic behaviour has been employed. All the systems considered in this work belong to the C_2_ point group, the z axis being the binary rotation axis, such symmetry has been exploited in the calculations. Core electrons have been kept frozen up to the Au 4f level. Relativistic effects have been considered at scalar level employing the zero order regular approximation (ZORA) level [38].

The TDDFT equations have been solved by means of the polTDDFT method [30,31] assuming adiabatic local density approximation for the response XC kernel.

In the polTDDFT scheme we have divided the excitation energy in intervals with a step of 0.025 eV and a cutoff of 2 eV above the excitation energy has proven accurate. An imaginary part of 0.060 eV has been employed in the photon energy, corresponding to an intrinsic lorentzian broadening of the same HWHM value.

All the calculations have been performed with the AMS–ADF suite of programs [39].
